# Machine learning-selected minimal features drive high-accuracy rule-based antibiotic susceptibility predictions for *Staphylococcus aureus* via metagenomic sequencing

**DOI:** 10.1128/spectrum.00556-25

**Published:** 2025-07-11

**Authors:** Xuefeng Jia, Yongfen Xiong, Yanping Xu, Fangyuan Chen, Peng Han, Jieming Qu, Quanli He, Guanhua Rao

**Affiliations:** 1 Frontiers Science Center for Synthetic Biology and Key Laboratory of Systems Bioengineering (Ministry of Education), School of Chemical Engineering and Technology, Tianjin University, Tianjin, China; 2 Frontiers Research Institute for Synthetic Biology, Tianjin University, Tianjin, China; 3 Department of Medical Laboratory, Wuhan No.1 Hospital, Wuhan, China; 4 Department of Pulmonary and Critical Care Medicine, Ruijin Hospital, Shanghai Jiao Tong University School of Medicine, Shanghai, China; 5 Institute of Respiratory Diseases, Shanghai Jiao Tong University School of Medicine 56694, Shanghai, China; 6 Shanghai Key Laboratory of Emergency Prevention, Diagnosis and Treatment of Respiratory Infectious Diseases, Shanghai, China; 7 Genskey Medical Technology Co., Ltd, Beijing, China; 8 Department of Clinical Laboratory, The People’s Hospital of Jiaozuo, Jiaozuo, China; UCI Health, Orange, California, USA

**Keywords:** metagenomic sequencing, *Staphylococcus aureus*, machine learning, rule-based model, antibiotic susceptibility predictions, vancomycin

## Abstract

Antimicrobial resistance (AMR) represents a critical global health challenge, demanding rapid and accurate antimicrobial susceptibility testing (AST) to guide timely treatments. Traditional culture-based AST methods are slow, while existing whole-genome sequencing (WGS)-based models often suffer from overfitting, poor interpretability, and diminished performance on clinical metagenomic data. In this study, we developed an interpretable genotypic AST approach for *Staphylococcus aureus* using minimal genomic determinants. Analysis of 4,796 *S*. *aureus* genomes and AST data for 18 antibiotics revealed one to five key resistance genes per antibiotic, including two previously uncharacterized vancomycin resistance markers. These features enabled highly accurate rule-based predictions, achieving area under the curve (AUC) values ranging from 0.94 to 1.00. The model demonstrated an overall sensitivity of 97.43% and specificity of 99.02%, respectively, with a very major error (VME) rate of 2.57% and a major error (ME) rate of 0.98% for isolate-level testing. Furthermore, after optimization for shallow-depth metagenomic sequencing, the model achieved 81.82% to 100% accuracy in AST predictions for 59 clinical samples, bypassing the need for bacterial isolation and reducing diagnostic time by an average of 39.9 hours. By combining minimal feature selection with strong interpretability and adaptability to metagenomic data, this method offers a practical and transformative solution for rapid and reliable AST in clinical settings.

**IMPORTANCE:**

Antimicrobial resistance (AMR) in *Staphylococcus aureus* poses a critical challenge to global health, necessitating rapid and reliable antimicrobial susceptibility testing (AST) for timely treatment decisions. Traditional culture-based AST is slow, while existing whole-genome sequencing (WGS)-based approaches often suffer from overfitting and poor interpretability. This study introduces a rule-based, interpretable genotypic AST model for *S. aureus* that leverages minimal genomic determinants, achieving over 97% accuracy in isolate-level testing and high accuracy in clinical metagenomic samples. By extracting key resistance features and applying a rule-based approach, our model enables faster AST predictions and enhances hospital surveillance of resistant strain outbreaks. This culture-independent method reduces diagnostic time by nearly 40 hours, providing a scalable and actionable solution for clinical AMR management.

## INTRODUCTION

Antimicrobial resistance (AMR) has emerged as one of the most significant global public health threats. A systematic analysis in 2019 estimated that bacterial AMR caused 1.27 million deaths and contributed to nearly 5 million deaths worldwide (1). Among the pathogens driving this burden, *Staphylococcus aureus* stands out as a major contributor due to its resistance to multiple antibiotics, including beta-lactams and glycopeptides. Methicillin-resistant *S. aureus* (MRSA) and vancomycin-resistant *S. aureus* (VRSA) have become endemic in hospitals globally, resulting in higher treatment costs, prolonged hospitalizations, and poorer outcomes (2). Infections caused by intermediate and resistant strains, such as heterogeneously vancomycin-intermediate *S. aureus* (hVISA), further exacerbate these challenges by increasing treatment failure rates (3). These trends underscore the urgent need for rapid and accurate antimicrobial susceptibility testing (AST) to enable early and targeted therapy, ultimately improving patient outcomes and mitigating the global AMR burden (4).

Current AST methods face significant limitations. Culture-based AST, considered the gold standard, typically requires 48–72 hours to provide results, often delaying the initiation of effective antibiotic therapy. This delay contributes to suboptimal patient outcomes, particularly in critical conditions such as sepsis, where each hour of delay is linked to increased mortality (5, 6). Empirical therapy, necessitated by these delays, proves inadequate in 25% of cases and ineffective in 8–12% of patients (7). Alternative rapid AST methods, such as phenotypic approaches using bacterial surface labeling or molecular-based genotypic approaches like PCR and whole-genome sequencing (WGS), offer some improvements but come with their own challenges. Phenotypic methods often require prior species identification, limiting their applicability in polymicrobial infections (4). Genotypic methods, while promising, typically depend on bacterial culture or enrichment, delaying results and complicating applications for slow-growing or hard-to-culture pathogens (8, 9).

To address these challenges, we developed a rule-based approach employing metagenomic next-generation sequencing (mNGS) to predict antibiotic susceptibility for *Staphylococcus aureus* directly from clinical samples. Unlike whole-genome sequencing (WGS) or culture-based methods, mNGS enables the unbiased analysis of complex microbial communities without prior enrichment, thereby substantially reducing turnaround time. Our method integrates open reading frame (ORF)-based prediction and machine learning to identify key genomic determinants for each pathogen-antibiotic pair. To enhance robustness and interpretability, clustering algorithms were applied to group key features, and a curated resistance gene database, GenseqResDB, was constructed with improved annotation accuracy. Leveraging this refined database, we developed rule-based models for antibiotic susceptibility prediction. Using simulated short-read data at varying sequencing depths, we systematically evaluated the performance of these models and identified sequencing depth thresholds required for reliable reporting of resistance and susceptibility. This framework ensured an accurate transition from WGS-AST models to mNGS-AST models. Validation using clinical specimens demonstrated the model’s ability to deliver highly accurate predictions even at lower sequencing depths. By enabling rapid, interpretable, and clinically actionable antimicrobial susceptibility testing, this approach represents a significant advancement over traditional culture-based methods, offering a promising solution to accelerate the diagnosis of antimicrobial resistance in clinical practice.

## MATERIALS AND METHODS

### Collection of bacterial genomes and AST data

We sourced *Staphylococcus aureus* genomes along with their corresponding antimicrobial susceptibility testing (AST) data from publicly accessible databases, including BV-BRC and NDARO, as well as from local hospital collections (Table S1). A stringent quality control process was implemented to exclude genomes of suboptimal quality. To ensure high-quality *Staphylococcus aureus* genomes, we applied customized criteria based on NCBI genome exclusion rules to discard low-quality genomes. The criteria for exclusion included: (a) inconsistent AST conclusions; (b) N50 <5000 bp or more than 2,000 contigs; (c) assembled genome length deviating by 1.5 times longer or 0.5 times shorter than the average for the species; (d) predicted gene count being 0.5 times lower than the average or not within the range of 0.5 to 1.5 times the expected CDS per 1000 bp; (e) average nucleotide identity (ANI) <0.95 or inconsistent taxonomy annotation when aligning contigs to the NCBI NT database; and (f) absence of 16S rDNA (10). To investigate the genetic diversity of *Staphylococcus aureus* populations, a phylogenetic tree was constructed based on the single-copy core genes of *Staphylococcus aureus*. First, the coding genes for each strain were predicted using GeneMarkS software (Version 4.17, http://topaz.gatech.edu/GeneMark). Subsequently, core genes were analyzed using clustering methods with CD-HIT (Version 4.6.1), with a threshold set at 50% pairwise identity and a 0.7 amino acid length difference cutoff to identify single-copy genes and align the core genes. A phylogenetic tree was constructed using the maximum likelihood model with Treebest (Version 1.9.2). Finally, the phylogenetic tree was visualized and enhanced using iTOL (https://itol.embl.de), with annotations for the antibiotic sensitivity test conclusions (11).

### Feature selection for AMR prediction using machine learning

To identify antimicrobial resistance (AMR) features, two distinct strategies were employed prior to constructing a genotype-phenotype association model using machine learning algorithms. The first approach, termed the “CARD-based strategy,” involved aligning genome contigs to the CARD database, which provides high-confidence annotations for antimicrobial resistance genes (ARGs) (12). The second approach, referred to as the “ORF-based strategy,” screened open reading frames (ORFs) to identify potential resistance features not covered by the CARD database. The CARD-based strategy was prioritized due to the robustness of its annotations. However, for antibiotics lacking representation in CARD, the ORF-based strategy was utilized to uncover additional candidate resistance determinants.

For each pathogen-antibiotic pair, the positive predictive value (PPV) of each candidate feature was calculated using the training data set, and features with low predictive power (e.g., PPV <0.8) were excluded. A lasso regression model with tenfold cross-validation was applied to identify key AMR features by minimizing the coefficient of variation (CV) error (13). The final model’s performance was assessed using the area under the receiver operating characteristic curve (AUC). Additionally, model stability was evaluated by analyzing performance variations across randomly subsampled data sets.

### Development of GenseqResDB

GenseqResDB, a customized ARG reference database, was constructed by expanding upon our previous work. The database integrates ARG sequences from the CARD database (version 3.1.0) and categorizes resistance determinants into six hierarchical levels: sequence type, gene type, subfamily, family, mechanism, and class. Newly identified resistance genes and subtypes absent from CARD were incorporated and annotated with their likely species of origin. Crucially, the database also establishes pathogen–antibiotic–resistance feature associations identified through machine learning and manually curated for accuracy.

### AMR feature identification and resistance prediction using GenseqAMR

GenseqAMR, a genotypic AMR prediction tool, was designed to detect AMR features in clinical samples and infer resistance or susceptibility by aligning sequencing reads obtained via metagenomic sequencing (mNGS). A greedy algorithm coupled with localized alignment filters was employed to minimize false-positive identifications of ARGs. For resistance prediction, a weighted scoring system was used:


$$Score=\sum_{i=1}^{n}{AMR\_feature\_W}_{i}$$

where *AMR_feature_W* represents the weight assigned to each detected resistance feature and *n* denotes the total number of features identified. Predictions were made by comparing the calculated score to a predefined cutoff value.

### Simulation experiments

Simulated short-read sequencing data for clinical isolates were generated using ART software (version 2.5.8) to evaluate GenseqAMR. These simulations assessed (i) the accuracy of AMR feature detection, (ii) the impact of sequencing depth on prediction performance, and (iii) the accuracy of origin species attribution. Optimal cutoff values and reporting criteria were determined through these analyses.

### Validation with retrospective clinical samples

Between September 2020 and September 2023, 59 clinical specimens from suspected infection cases were collected at Wuhan No.1 Hospital, Ruijin Hospital, Shanghai Jiao Tong University, and Jiaozuo People’s Hospital. Ethical approvals were obtained from the institutional review board (RJ2019NO1-3). Microbial identification for culture-based isolates was conducted using MALDI-TOF MS (Bruker Microflex) with identification scores > 2.0, while mNGS data were generated using Genskey Medical Technology’s sequencing platform. A previously described bioinformatics pipeline was used for pathogen identification (14), and GenseqAMR was applied to predict resistance phenotypes. Model performance metrics, including PPV, NPV, and the proportion of reportable samples, were calculated. The time required for AST results via culture-based methods was compared to that of GenseqAMR.

## RESULTS

### Screening antimicrobial resistance determinants in whole genomes using machine learning

**Fig 1:**
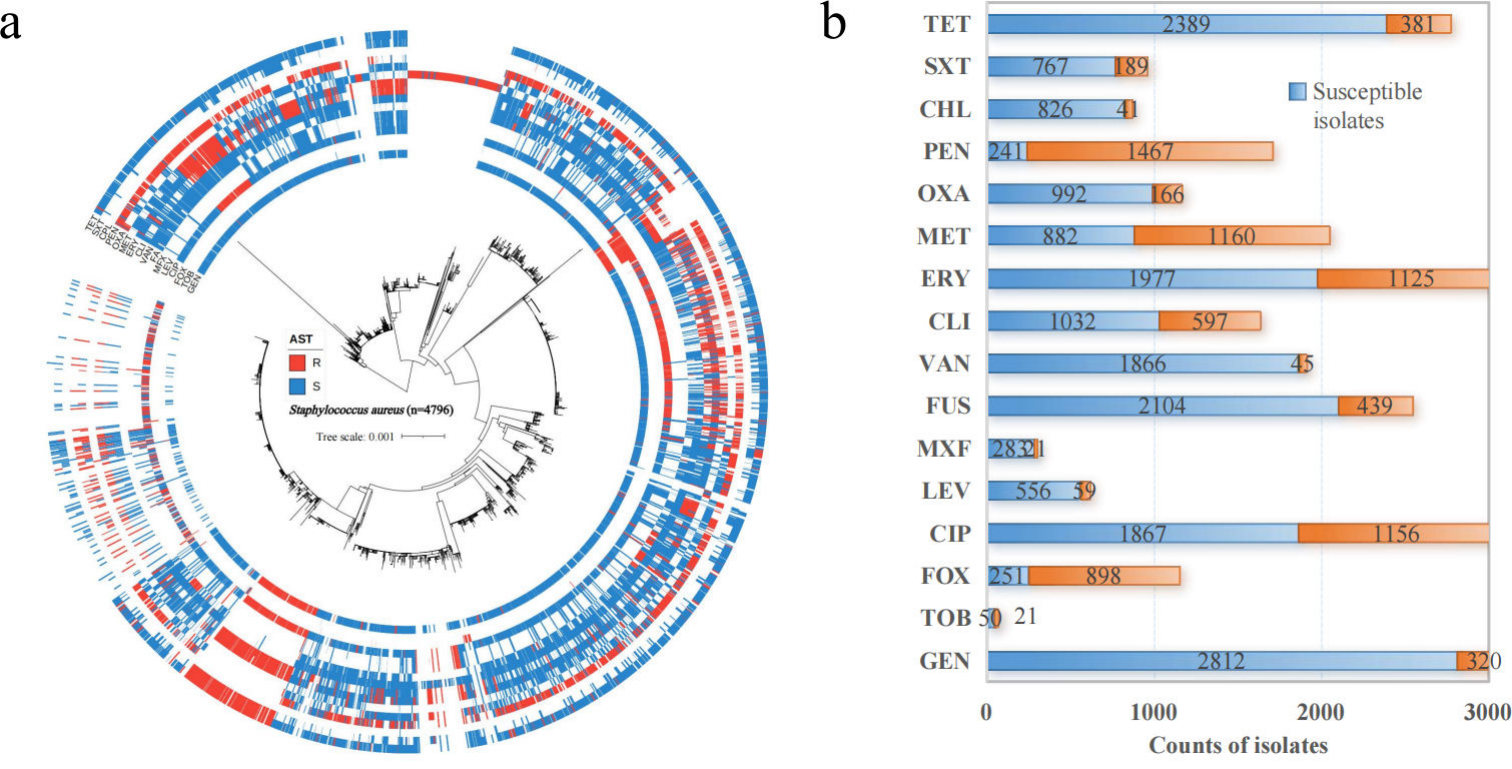
Description of the *Staphylococcus aureus* genome collection. (a) Phylogenetic tree with annotation of AST results. The maximum likelihood phylogenetic tree was constructed based on the core genes and plotted for *S. aureus* using iTOL (https://itol.embl.de) with annotation of AST conclusions in the outer circles. The red bar means resistance, while the blue is susceptibility. (b) Number of tested isolates for each antibiotic. GEN, gentamicin; KAN, kanamycin; TOB, tobramycin; FOX, cefoxitin; CIP, ciprofloxacin; LVX, levofloxacin; MXF, moxifloxacin; ERY, erythromycin; CLI, clindamycin; VAN, vancomycin; SXT, trimethoprim_sulphamethoxazole; TET, tetracycline; MET, methicillin; OXA, oxacillin; PEN, penicillin; CHL, chloramphenicol; FUS, fusidic_acid; MUP, mupirocin.

After quality control and data filtering, we gathered the whole genomes and antimicrobial resistance (AMR) profiles of 4,796 *S*. *aureus* isolates obtained from the Bacterial and Viral Bioinformatics Resource Center (BV-BRC), the NCBI National Database of Antibiotic Resistant Organisms (NDARO), and local hospitals in China (Table S1). Phylogenetic analysis of these bacterial genomes, based on core genes, revealed significant diversity, as depicted by the maximum likelihood phylogenetic trees (Fig. 1a). Except for tobramycin (TOB) and kanamycin (KAN), which had only 71 and 64 isolates, respectively, all other pathogen-antibiotic pairs included a sufficient number of resistant and susceptible isolates (Fig. 1b).

**Fig 2:**
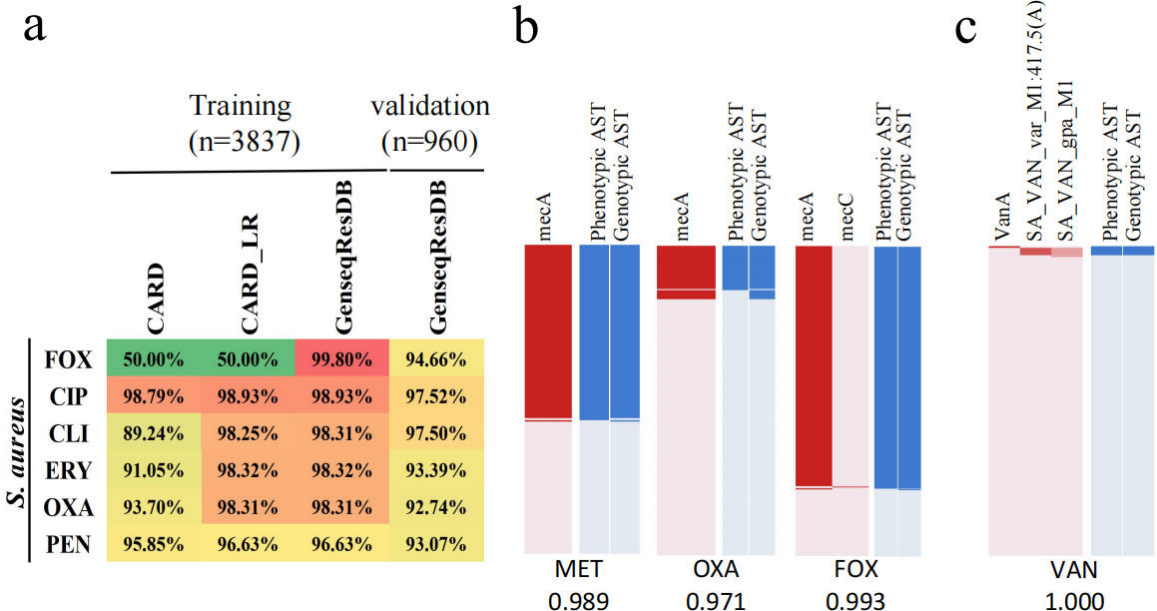
The consistency between genotype and phenotype in bacterial antibiotic resistance. (**a)** The performance of WGS-AST models constructed based on three different strategies—CARD, CARD_LR, and GenseqReDB. CARD strategy, AST prediction based on the ARGs with the annotation conferring to antibiotic resistance from the CARD database. CARD_LR, AST prediction based on the integrated AMR features obtained from CARD strategy and lasso regression model. GenseqResDB, AST prediction based on the integrated AMR features obtained from CARD strategy or ORF-based method with lasso regression model and manual correction or recall of key low-frequency features. (b and c) Key antimicrobial resistance (AMR) features for different antibiotic screenings. In each subplot, each row represents a strain. The left panel displays the distribution of detected features, with red indicating detection and light pink indicating non-detection. The right panel shows the results of antibiotic susceptibility testing (AST) based on culture and genotype-based resistance predictions, where blue indicates resistance and light blue indicates susceptibility. The AUC values for each antibiotic are marked in the bottom area. MET, methicillin; OXA, oxacillin; FOX, cefoxitin; VAN, vancomycin. SA_VAN_var_M1 and SA_VAN_gpa_M1 are characteristic genes identified through the “ORF-based strategy.”

To link genetic factors with AMR phenotypes, we focused on identifying highly relevant genetic features and assessing their predictive power when used as input data. Following the methodology outlined in our previous research, we utilized the lasso regression algorithm to analyze genotype–phenotype relationships for each pathogen-antibiotic pair. When comparing the use of direct alignment with known antibiotic resistance genes (ARGs) and mutational variants from the Comprehensive Antibiotic Resistance Database (CARD), the predictive performance of several models significantly improved after applying lasso regression for feature selection (Fig. 2a). However, no significant improvement was observed in the performance of the cefoxitin model.

Previously, we reported an ORF-based method for identifying resistance features, which proved effective in optimizing colistin resistance predictions in *Escherichia coli*. In the present study, we extended this approach to refine novel resistance features and integrated these with an enhanced public resistance database to construct a curated ARG reference database, GenseqResDB, designed to enhance predictive performance. Rule-based WGS-AST models built using this database consistently achieved AUC values exceeding 0.94 (Table 1). Key resistance features associated with 18 antibiotics were identified as shown in Fig. 2b and c and Fig. S1a through n, and the contribution of each resistance marker to the lasso regression classifier for different antibiotics is detailed in Table S2. The AUC of the cefoxitin model improved markedly from 0.5 to 0.98, largely due to the identification of mecA as a critical determinant of cefoxitin resistance in *Staphylococcus aureus* (15). However, in the CARD database, mecA was associated exclusively with resistance to methicillin and oxacillin, but not to cefoxitin (Fig. 2b).

**TABLE 1:** Performance of the lasso regression classifier to predict *S. aureus* susceptibility or resistance to 16 antibiotics^*a*^

Antibiotics	Total no. of isolates	True positive	False positive	True negative	False negative	Sensitivity	Specificity	PPV	NPV	VME	ME	Accuracy	AUC	No. of ARGs	Key ARGs
Gentamicin	3,132	304	10	2,802	16	95% (304/320)	99.64% (2,802/2,812)	96.82% (3,04/314)	99.43% (2,802/2,818)	5% (16/320)	**0.36%** (**10/2,812**)	99.17% (3,106/3,132)	0.97	1	AAC(6')-Ie-APH(2'')-Ia
Tobramycin	71	21	0	50	0	100% 21/21)	100% (50/50)	100% (21/21)	100% (50/50)	**0%** (**0/21**)	**0%** (**0/50**)	100% (71/71)	1.00	1	AAC(6')-Ie-APH(2'')-Ia
Cefoxitin	1,149	896	5	246	2	99.78% (896/898)	98.01% (246/251)	99.45% (896/901)	99.19% (246/248)	**0.22%** (**2/898**)	**1.99%** (**5/251**)	99.39% (1,142/1,149)	0.99	2	mecA; mecC
Ciprofloxacin	3,023	1136	11	1,856	20	98.27% (1,136/1,156)	99.41% (1,856/1,867)	99.04% (1,136/1,147)	98.93% (1,856/1,876)	1.73% (20/1156)	**0.59%** (**11/1867**)	98.97% (2,992/3,023)	0.99	2	SA_parC:239(C->T/A)/250(G->A)/251(A->G); SA_gyrA:250(T->G)/251(C->T)/253(T->C)
Levofloxacin	615	57	0	556	2	96.61% (57/59)	100% (556/556)	100% (57/57)	99.64% (556/558)	3.39% (2/59)	**0%** (**0/556**)	99.67% (613/615)	0.98	1	SA_gyrA:250(T->G)/251(C->T)
Moxifloxacin	304	21	0	283	0	100% (21/21)	100% (283/283)	100% (21/21)	100% (283/283)	**0%** (**0/21**)	**0%** (**0/283**)	100% (304/304)	1.00	1	SA_gyrA:251(C->T)
Fusidic_acid	2,543	422	7	2,097	17	96.13% (422/439)	99.67% (2,097/2,104)	98.37% (422/429)	99.20% (2,097/2,114)	3.87% (17/439)	**0.33%** (**7/2,104**)	99.06% (2,519/2,543)	0.98	3	FusC; FusB; FusA:268(G->A)/1211(C->A/T)/1369(C->T)/1371(C->A/G)/1381(T->A)/1382(T->C/A)
Vancomycin	1,911	45	0	1,866	0	100% (45/45)	100% (1,866/1,866)	100% (45/45)	100% (1,866/1,866)	**0%** (**0/45**)	**0%** (**0/1,866**)	100% (1,911/1,911)	1.00	2	SA_VAN_var_M1:417.5(A) (SRPBCC domain-containing protein); SA_VAN_gpa_M1 (degv, DegV domain-containing protein)
Clindamycin	1,629	577	18	1,014	20	96.65% (577/597)	98.26% (1,014/1,032)	96.97% (577/595)	98.07% (1,014/1,034)	3.35% (20/597)	**1.74%** (**18/1,032**)	97.67% (1,591/1,629)	0.98	4	ErmA; ErmB; ErmC; ErmT
Erythromycin	3,102	1,086	21	1,956	39	96.53% (1,086/1,125)	98.94% (1,956/1,977)	98.1% (1,086/1,107)	98.05% (1,956/1,995)	3.47% (39/1,125)	**1.06%** (**21/1,977**)	98.07% (3,042/3,102)	0.98	5	ErmA; ErmB; ErmC; ErmT; msrA
Methicillin	2,042	1,146	8	874	14	98.79% (1,146/1,160)	99.09% (874/882)	99.31% (1,146/1,154)	98.42% (874/888)	**1.21%** (**14/1,160**)	**0.91%** (**8/882**)	98.92% (2,020/2,042)	0.99	1	mecA
Oxacillin	1,158	162	34	958	4	97.59% (162/166)	96.57% (958/992)	82.65% (162/196)	99.58% (958/962)	2.41% (4/166)	3.43% (34/992)	96.72% (1,120/1,158)	0.97	1	mecA
Penicillin	1,708	1,445	20	221	22	98.5% (1,445/1,467)	91.7% (221/241)	98.63% (1,445/1,465)	90.95% (221/243)	**1.5%** (**22/1,467**)	8.3% (20/241)	97.54% (1,666/1,708)	0.96	2	mecA; blaZ
Chloramphenicol	867	37	0	826	4	90.24% (37/41)	100% (826/826)	100% (37/37)	99.52% (826/830)	9.76% (4/41)	**0%** (**0/826**)	99.54% (863/867)	0.95	3	Efae_ACT_CHL; Sint_ACT_CHL; Efac_ACT_CHL
Trimethoprim_ sulfamethoxazole	956	174	32	735	15	92.06% (174/189)	95.83% (735/767)	84.47% (174/206)	98% (735/750)	7.94% (15/189)	4.17% (32/767)	95.08% (909/956)	0.94	1	dfrG
Tetracycline	2,770	375	19	2,370	6	98.43% (375/381)	99.2% (2,370/2,389)	95.18% (375/394)	99.75% (2,370/2,376)	1.57% (6/381)	**0.8%** (**19/2,389**)	99.1% (2,745/2,770)	0.99	2	tet(K); tetM
Overall	26,980	7,904	185	18,710	181	97.76% (7,904/8,085)	99.02% (18,710/18,895)	97.71% (7,904/8,089)	99.04% (18,710/18,891)	2.24% (181/8085)	**0.98%** (**185/18895**)	98.64% (26,614/26,980)	/	/	/

^*a*^ Bold entries denote VME values below 1.5% and ME values below 3%, meeting the requirement set by the Food and Drug Administration (FDA).

Besides the previously reported vanA gene, we also discovered novel resistance markers for vancomycin in *S. aureus*, including SA_VAN_var_M1:417.5(A), encoding a protein with a SRPBCC domain, and SA_VAN_gpa_M1 (degv, a DegV domain-containing protein). These features yielded positive predictive values (PPVs) of 0.978 (45/46) and 0.804 (45/56), respectively. Incorporating these features into the vancomycin WGS-AST prediction model produced an AUC of 1.0, underscoring their potential clinical relevance (Fig. 2c; Table S2). We next explored the impact of sample size on model stability. As demonstrated in Fig. S2, the performance of classification models reached saturation well before utilizing the full data set, indicating that adding more isolates yields only marginal improvements in classification accuracy.

### Development of an mNGS-AST prediction model

**Fig 3:**
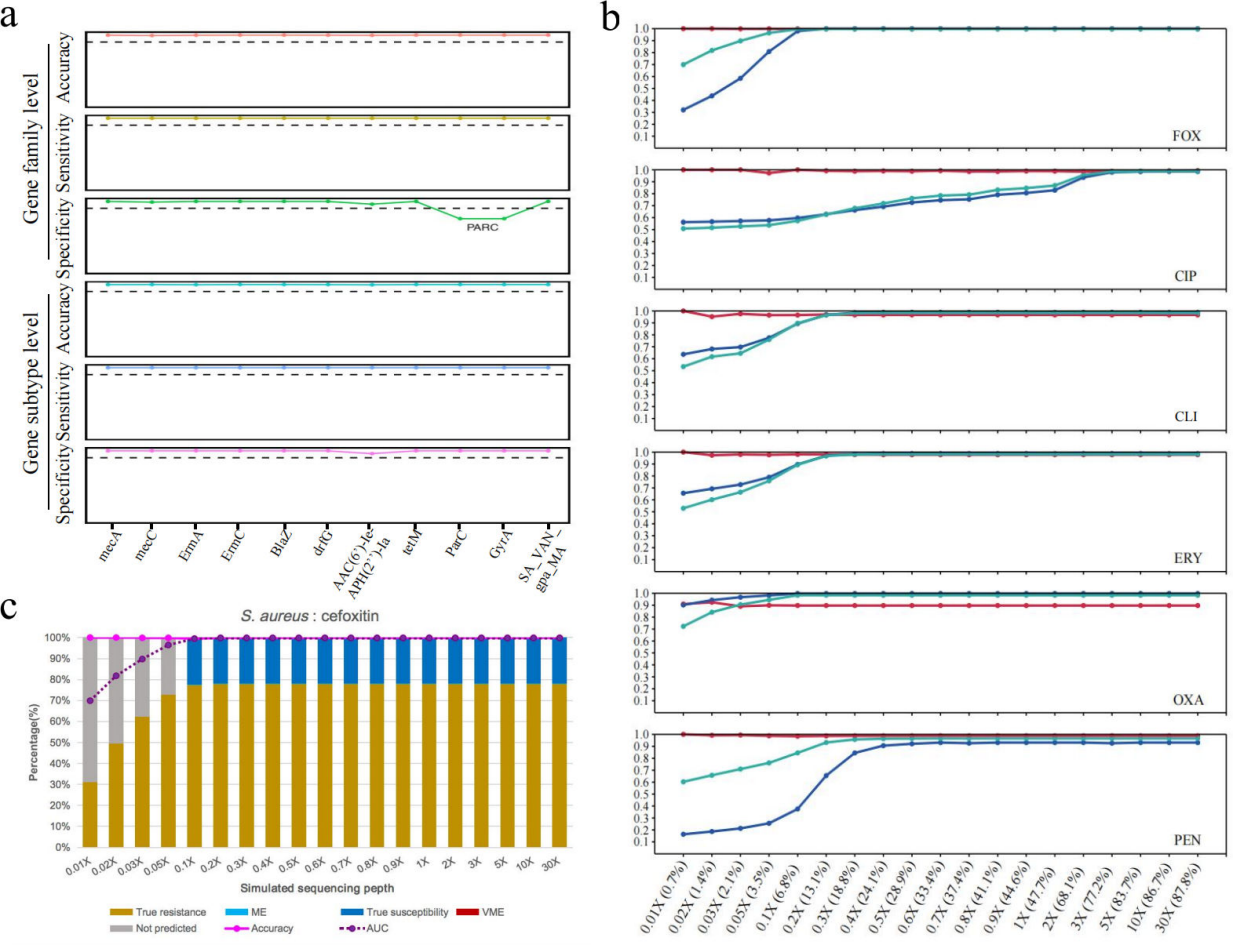
Performance of genotypic AST prediction model on different sequencing depth of bacterial genome. (a) The sensitivity, specificity, and accuracy of read-based ARG subtype and ARG family test for *S. aureus*. The black dotted line is 0.9. The horizontal axis represents each important ARG screened. (b) The read-based model performance of six antibiotics at different sequencing depths through simulation experiment. The horizontal axis represents the simulated sequencing depth and truly detected genome coverage separated by a colon. The red line, PPV; the blue line, NPV; the green line, AUC. (c) Performance of the mNGS-AST prediction model at different simulated sequencing depths for *S. aureus*-cefoxitin. The horizontal axis represents the simulated genomic sequencing depth. The purple dashed line indicates the AUC value of the model at different simulated genomic sequencing depths. The magenta line shows the prediction accuracy of the mNGS-AST model at different sequencing depths based on well-defined reporting rules. The bar graphs refer to the proportions of samples with correct, incorrect, and unreportable predictions. True resistance, both phenotypic AST and genotypic prediction, indicates resistance. ME, major error, phenotypic AST indicates susceptibility, while genotypic prediction indicates resistance. True susceptibility, both phenotypic AST and genotypic prediction, indicates susceptibility. VME, very major error, phenotypic AST indicates resistance, while genotypic prediction indicates susceptibility.

The clinical application of WGS-AST is limited to culture-positive samples; however, metagenomic sequencing (mNGS) could bypass the need for culturing and enable direct analysis of clinical specimens. The key challenge lies in adapting the WGS-AST prediction model into an mNGS-AST model, referred to here as the GenseqAMR model, while maintaining robust performance. Given the characteristics of mNGS data, such as short reads and shallow sequencing depth of target pathogens, we first assessed whether the established pipeline could effectively identify previously determined key resistance features using 50 bp short reads. To simulate mNGS data, we selected 11 key resistance features and generated synthetic data sets with a sequencing depth of 30×. Compared to genome assembly data, GenseqAMR demonstrated sensitivity, specificity, and accuracy exceeding 0.9 in tests for ARG subtypes and ARG families of *S. aureus* (Fig. 3a). This suggests that changes in read length alone have minimal impact on the accurate identification of key ARGs in *S. aureus*.

We next evaluated the effect of sequencing depth on predictive accuracy. As expected, the predictive performance of GenseqAMR improved with increasing sequencing depth and eventually plateaued (Fig. 3b). For prediction models based on the presence or absence of genes rather than gene subtypes, the minimum sequencing depth required for near-maximal performance was typically 0.2×, 0.3×, or 0.5×. In contrast, models utilizing genetic mutations or gene subtypes required a higher minimum sequencing depth of up to 3×. Due to the variable sequencing depths for target pathogens in mNGS data, there is a potential risk of failing to detect key resistance genes at lower sequencing depths, leading to false-negative results. To address this, we defined the minimum genome coverage necessary for reliable susceptibility predictions. This threshold ensures that susceptibility predictions are only reported when sequencing depth is sufficient to rule out false negatives. For instance, if the sequencing depth of the *S. aureus* genome is below 0.2 × and no AMR markers are detected, susceptibility to cefoxitin cannot be predicted (Fig. 3c). The minimum genome coverage thresholds required for predicting susceptibility to other antibiotics are detailed in Table S3.

### Performance validation in retrospective case studies

**Fig 4:**
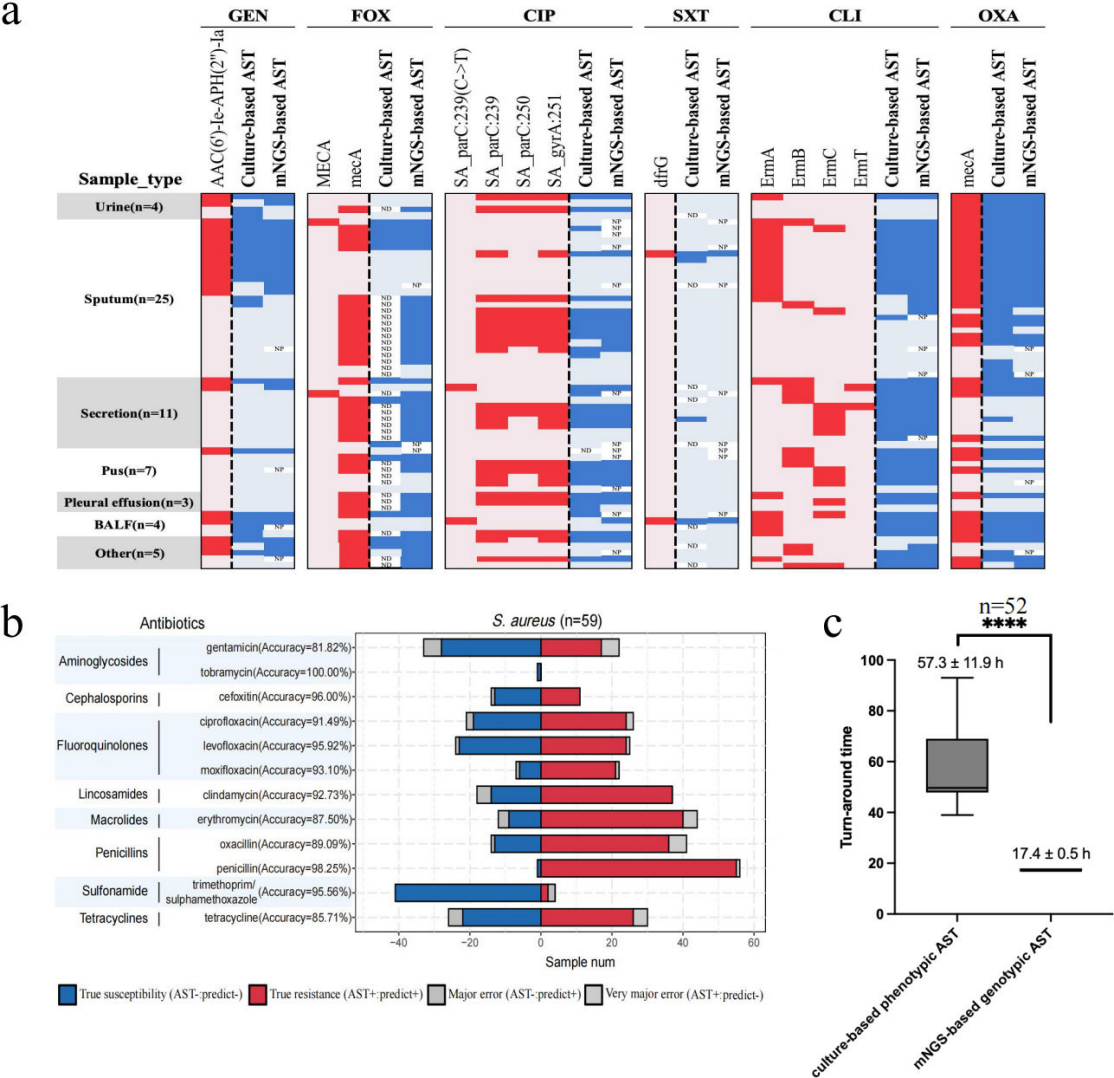
Direct application of mNGS-based AST for clinical specimens. (a) The graph displays a heatmap of read-based GenseqAMR predictions for different antibiotics in 59 *S*. *aureus* culture-positive clinical specimens. In each subgraph, the detected key antimicrobial resistance (AMR) features are shown on the left, with red indicating detected and light pink indicating not detected. On the right, the antimicrobial susceptibility test (AST) results from culture-based AST (VITEK-2 system) and mNGS-based AST are presented, with blue representing resistance and light blue representing susceptibility. ND denotes “Not Detected,” and NP denotes “Not Predicted.” (b) Validation of mNGS-based AST prediction models using retrospective clinical specimens. In each subplot, the left bar is the number of samples which are phenotypically susceptible while the right is that of resistant samples. (c) Perform a *t*-test analysis of the turnaround time for mNGS-based AST and culture-based phenotypic AST. **** represents *P* < 0.0001 (*t*-test). GEN, gentamicin; FOX, cefoxitin; CIP, ciprofloxacin; SXT, trimethoprim_sulphamethoxazole; CLI, clindamycin; OXA, oxacillin.

To evaluate the performance of the optimized mNGS-AST model on clinical mNGS data sets, we retrospectively analyzed 59 *S*. *aureus* culture-positive clinical samples. These included four urine, 25 sputum, 11 pus, seven secretions, three pleural effusions, four BALF, and five samples of other types. The detected AMR features and prediction results for each clinical specimen are detailed in Fig. 4a; Fig. S3 and Table S4.

Across the 59 clinical samples, the *S. aureus* genome coverage ranged from 0.96% to 96.59%. The average sensitivity, specificity, positive predictive value (PPV), and negative predictive value (NPV) of GenseqAMR predictions were 92.14%, 89.62%, 93.02%, and 88.37%, respectively. The overall accuracy was 91.13%, with a range of 81.82% to 100%, and the overall reportable rate was 91.07% (Fig. 4b; Table 2). Turnaround time comparisons revealed that GenseqAMR offered significantly shorter times (mean ± SD: 17.4 ± 0.5 hours) compared to traditional culture-based AST (mean ± SD: 57.3 ± 11.9 hours) (Fig. 4c; Table S5).

**TABLE 2:** Performance of the mNGS-AST model to predict susceptibility and resistance to 12 antibiotics in 59 clinical specimens

Antibiotics	AST+: predict+	AST-: predict+	AST+: predict-	AST-:predict-	AST_sample_num	Predict_sample_num	Accuracy	PPV	NPV	Predictable rate	Sensitivity	Specificity
Gentamicin	17	5	5	28	RS:59 (R:23 S:36-I:0)	RS:55 (R:22 S:33)	81.82% (45/55)	77.27% (17/22)	84.85% (28/33)	93.22% (55/59)	77.27% (17/22)	84.85% (28/33)
Tobramycin	0	0	0	1	RS:2 (R:1 S:1-I:0)	RS:1 (R:0 S:1)	100.00% (1/1)	NA (0/0)	100.00% (1/1)	50.00% (1/2)	NA (0/0)	100.00% (1/1)
Cefoxitin	11	1	0	13	RS:28 (R:12 S:16-I:0)	RS:25 (R:12 S:13)	96.00% (24/25)	91.67% (11/12)	100.00% (13/13)	89.29% (25/28)	100.00% (11/11)	92.86% (13/14)
Ciprofloxacin	24	2	2	19	RS:58 (R:27 S:29-I:2)	RS:47 (R:26 S:21)	91.49% (43/47)	92.31% (24/26)	90.48% (19/21)	81.03% (47/58)	92.31% (24/26)	90.48% (19/21)
Levofloxacin	24	1	1	23	RS:59 (R:27 S:31-I:1)	RS:49 (R:25 S:24)	95.92% (47/49)	96.00% (24/25)	95.83% (23/24)	83.05% (49/59)	96.00% (24/25)	95.83% (23/24)
Moxifloxacin	21	1	1	6	RS:30 (R:22 S:8-I:0)	RS:29 (R:22 S:7)	93.10% (27/29)	95.45% (21/22)	85.71% (6/7)	96.67% (29/30)	95.45% (21/22)	85.71% (6/7)
Clindamycin	37	4	0	14	RS:59 (R:39 S:20-I:0)	RS:55 (R:41 S:14)	92.73% (51/55)	90.24% (37/41)	100.00% (14/14)	93.22% (55/59)	100.00% (37/37)	77.78% (14/18)
Erythromycin	40	3	4	9	RS:59 (R:45 S:14-I:0)	RS:56 (R:43 S:13)	87.50% (49/56)	93.02% (40/43)	69.23% (9/13)	94.92% (56/59)	90.91% (40/44)	75.00% (9/12)
Oxacillin	36	1	5	13	RS:59 (R:43 S:16-I:0)	RS:55 (R:37 S:18)	89.09% (49/55)	97.30% (36/37)	72.22% (13/18)	93.22% (55/59)	87.80% (36/41)	92.86% (13/14)
Penicillin	55	0	1	1	RS:59 (R:58 S:1-I:0)	RS:57 (R:55 S:2)	98.25% (56/57)	100.00% (55/55)	50.00% (1/2)	96.61% (57/59)	98.21% (55/56)	100.00% (1/1)
Trimethoprim_ sulfamethoxazole	2	0	2	41	RS:51 (R:4 S:47-I:0)	RS:45 (R:2 S:43)	95.56% (43/45)	100.00% (2/2)	95.35% (41/43)	88.24% (45/51)	50.00% (2/4)	100.00% (41/41)
Tetracycline	26	4	4	22	RS:59 (R:30 S:29-I:0)	RS:56 (R:30 S:26)	85.71% (48/56)	86.67% (26/30)	84.62% (22/26)	94.92% (56/59)	86.67% (26/30)	84.62% (22/26)
Overall	293	22	25	190	582	530	91.13% (483/530)	93.02% (293/315)	88.37% (190/215)	91.07% (530/582)	92.14% (293/318)	89.62% (190/212)

## DISCUSSION

Antimicrobial resistance (AMR) poses a critical global health challenge, demanding rapid and accurate antimicrobial susceptibility testing (AST) to guide treatment and improve patient outcomes. Recent advancements in whole-genome sequencing (WGS) have enabled machine learning-based AMR phenotype predictions with high sensitivity and specificity for pathogens, such as *Mycobacterium tuberculosis* (16, 17), *Escherichia coli* (18–22), *Klebsiella pneumoniae* (23), *Pseudomonas aeruginosa* (24), *Staphylococcus aureus* (25, 26), and other species (8). Despite their promise, WGS-AST methods rely on cultured isolates, limiting their clinical utility in time-sensitive scenarios.

Our study addresses these limitations by bridging WGS-AST with metagenomic sequencing (mNGS), allowing direct application to clinical specimens. Building on prior work where we developed mNGS-based AMR models for *Acinetobacter baumannii* (10), *Pseudomonas aeruginosa* (27), and *Klebsiella pneumoniae* (11, 28), we now introduce an interpretable genotypic AST model for *Staphylococcus aureus*. Using 4,796 genomes, we identified minimal genomic determinants to predict resistance for 18 antibiotics. The model achieved exceptional isolate-level performance, with sensitivities of 97.43% (range: 95%–100%), specificities of 99.02% (range: 91.70%–100%), VME of 2.24% (range: 0–9.76%), and ME of 0.98% (range: 0–8.30%). Optimized for shallow-depth mNGS data, the model demonstrated 81.82%–100% accuracy in AST predictions across 59 clinical samples, bypassing the need for bacterial isolation and reducing diagnostic time by nearly 40 hours.

Our approach outperforms previous mNGS-AMR studies, which often relied on mapping short reads to databases like ResFinder or CosmosID. For instance, a proof-of-concept study achieved 94.1% accuracy in AMR prediction for as few as 24 culture-positive bone and joint infections and lacked generalizability to complex clinical samples (29). Similarly, Yan et al. reported sensitivities of 65.7%–85.0% for methicillin, clindamycin, and trimethoprim-sulfamethoxazole resistance prediction but struggled with incomplete genomic representation and false positives due to commensal organism contamination (30). These limitations highlight challenges in leveraging short-read mNGS for AMR detection, such as overestimated performance in culture-positive samples and inaccuracies stemming from uncontextualized AMR gene identification.

In contrast, our model focuses on a minimal set of robust genomic determinants validated for direct mNGS application. This design ensures high interpretability and reliability, overcoming overfitting and incomplete data issues. By achieving over 97% accuracy at the isolate level and maintaining strong performance in direct clinical samples, our work demonstrates the feasibility of mNGS-AST for real-world applications. Additionally, the discovery of novel vancomycin resistance markers underscores the model’s capacity to expand resistance insights.

Despite these advancements, several limitations of our study must be acknowledged. First, the clinical validation included a relatively small number of samples, and these were retrospectively collected, culture-positive specimens. This might limit the generalizability of our findings to broader patient populations, including culture-negative or more complex infection cases. Second, we did not evaluate the clinical benefits of our approach, such as its impact on patient outcomes, antibiotic stewardship, or cost-effectiveness. Future prospective studies with larger and more diverse cohorts, alongside clinical utility assessments, are essential to establish the broader applicability and value of this technology in routine diagnostics.

This study establishes a practical, scalable framework for mNGS-based AST, setting a new standard for integrating genotypic resistance prediction into routine diagnostics. Our findings provide a roadmap for transitioning from isolate-based WGS methods to rapid, culture-independent solutions, paving the way for improved AMR management in clinical care.

## Data Availability

Shotgun metagenomic sequencing data for 59 clinical specimens (with human DNA removed) have been deposited in the NCBI Sequence Read Archive under accession number PRJNA799652. The GenseqAMR pipeline, implemented using Perl, R, and sequence alignment tools such as ncbi-blast-2.9.0+ and minimap2 (version 2.17), is available upon request.

## SUPPLEMENTAL MATERIAL

Supplemental figures spectrum.00556-25-s0001.docx Figures S1 to S3.

Supplemental tables spectrum.00556-25-s0002.xlsx Tables S1 to S5.

ASM does not own the copyrights to Supplemental Material that may be linked to, or accessed through, an article. The authors have granted ASM a non-exclusive, world-wide license to publish the Supplemental Material files. Please contact the corresponding author directly for reuse.
