# Supplementary material for: Machine learning-selected minimal features drive high-accuracy rule-based antibiotic susceptibility predictions for Staphylococcus aureus via metagenomic sequencing
Source: Microbiol Spectr. 2025 Jul 11;13(8):e00556-25. doi: 10.1128/spectrum.00556-25 (PMC12323339; doi:10.1128/spectrum.00556-25)
Supplement: Supplemental figures — Figures S1 to S3. [file spectrum.00556-25-s0001.docx]

Supplementary Figures:


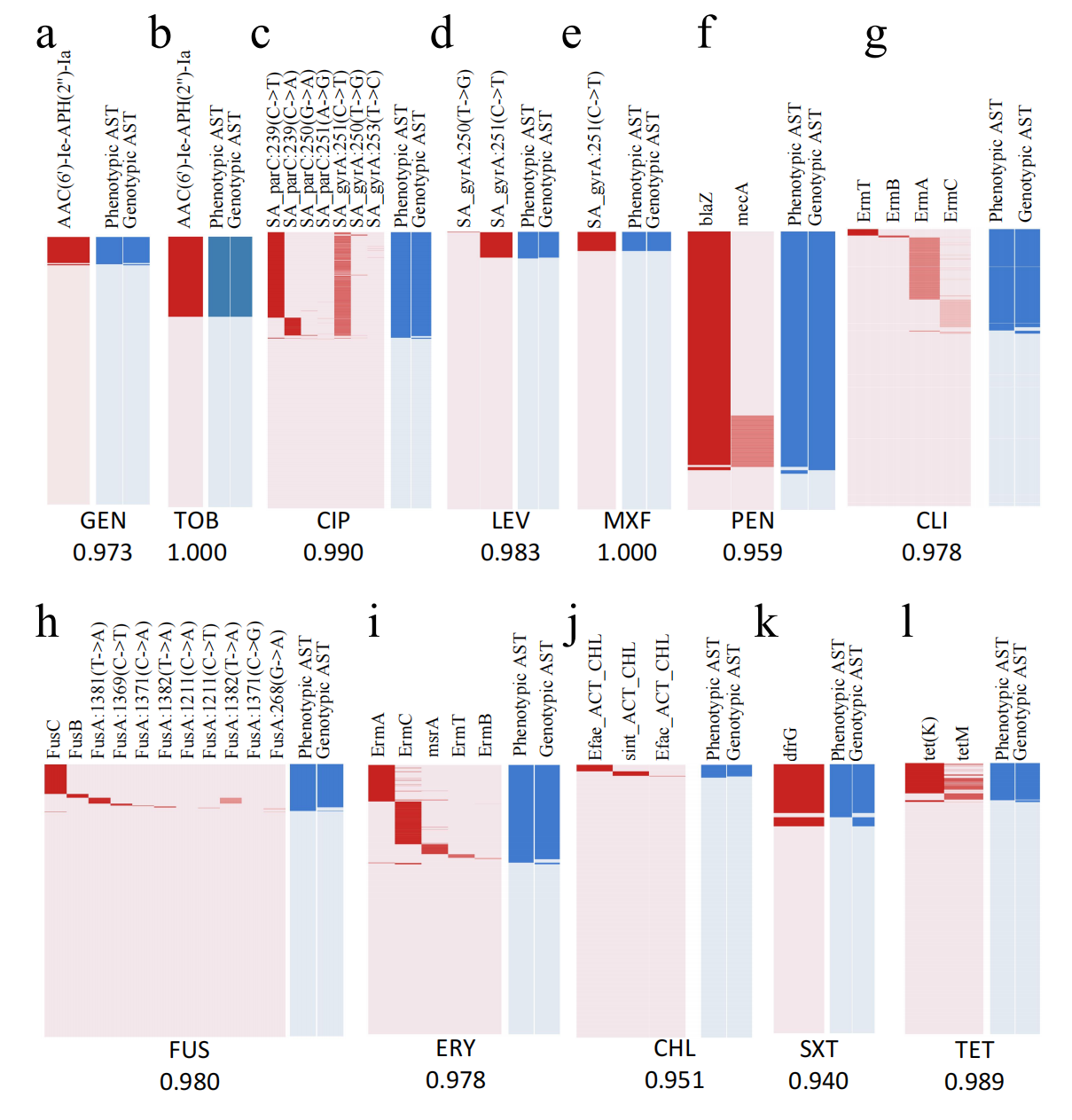


**Figure S1. key AMR features screened for different antibiotics.**

a-l, In each subgraph, the left panel shows the distribution of detected features while the right is the culture-based AST and genotypic AMR prediction results. The AUC value of each antibiotic was indicated at the bottom area. GEN, gentamicin;TOB, tobramycin; CIP, ciprofloxacin; LEV, levofloxacin; MXF, moxifloxacin; ERY, erythromycin; CLI, clindamycin; SXT, trimethoprim_sulphamethoxazole; TET, tetracycline;PEN, penicillin; CHL, chloramphenicol; FUS, fusidic_acid;


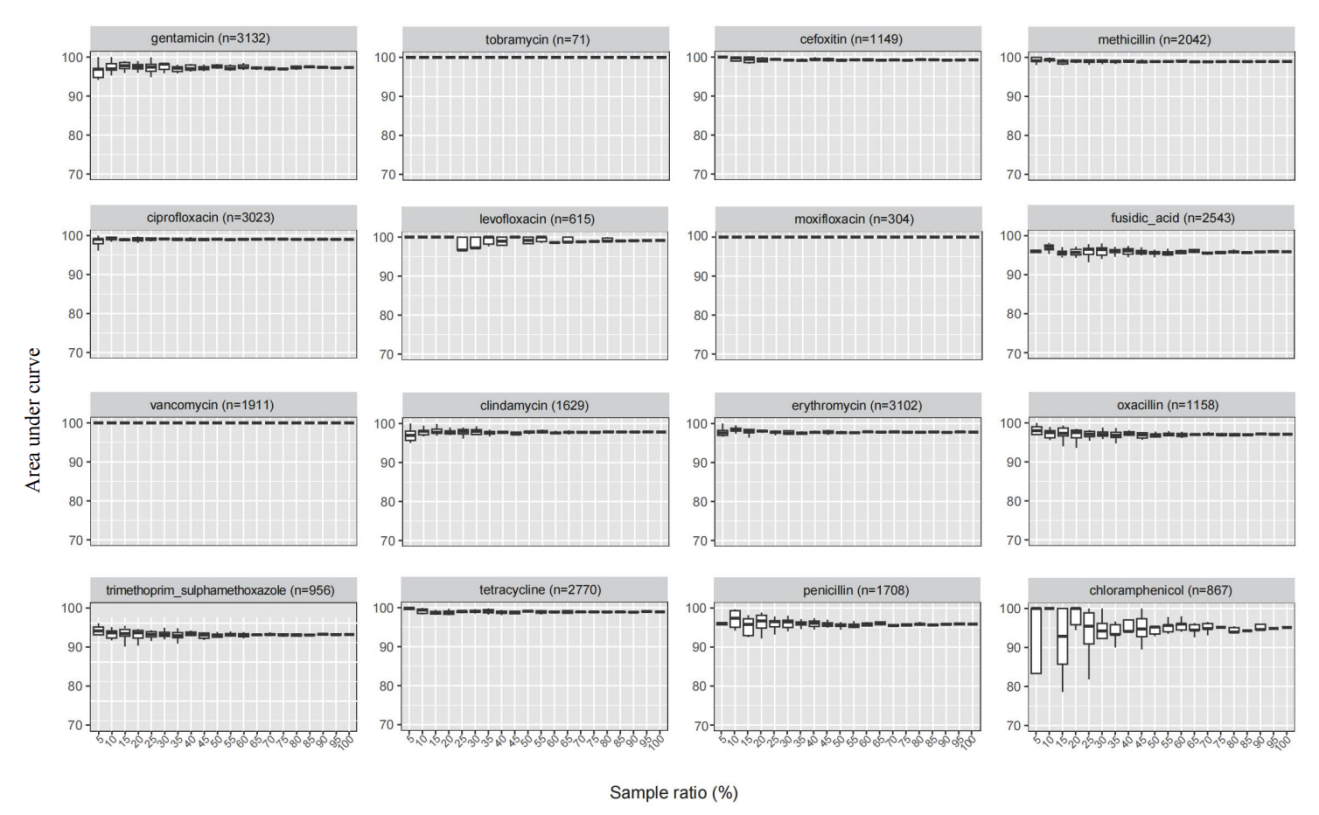


**Figure S2. Performance of WGS-AST prediction model under different subsamples for S. aureus.** A lasso regression model-based resistance/susceptibility classifier was trained on randomly drawn subsamples from the training dataset and evaluated in ten repeats of a 10-fold nested cross-validation. Each panel depicts the results of different antibitics based on the features of ARGs' presence/absence or variance.


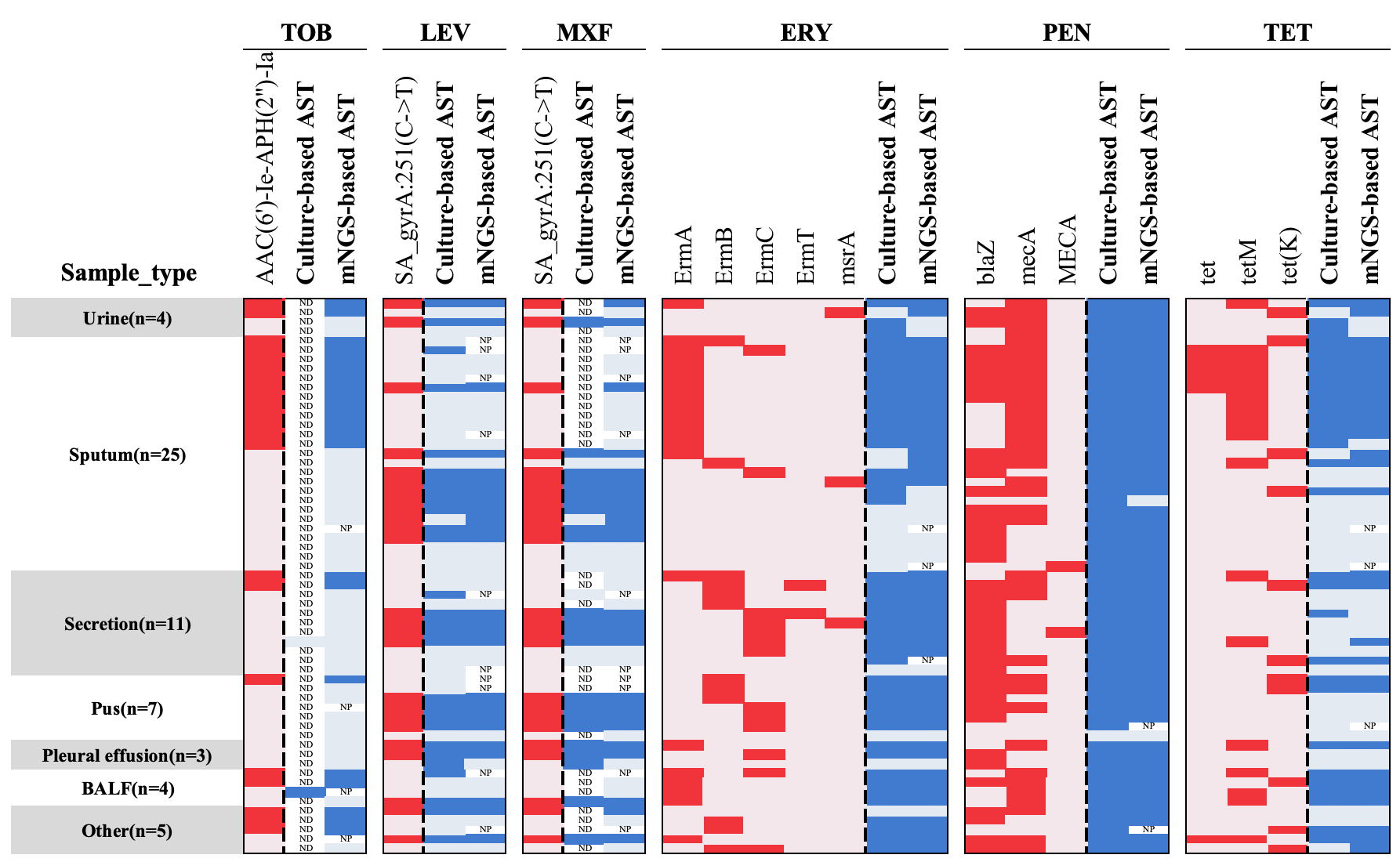


**Figure S3. Direct application of mNGS-based AST for clinical specimens.**

The graph displays a heatmap of read-based GenseqAMR predictions for different antibiotics in 59 S. aureus culture-positive clinical specimens. In each subgraph, the detected key antimicrobial resistance (AMR) features are shown on the left, with red indicating detected and light pink indicating not detected. On the right, the antimicrobial susceptibility test (AST) results from culture-based AST (VITEK-2 system) and mNGS-based AST are presented, with blue representing resistance and light blue representing susceptibility. ND represents "Not Detected," and NP represents "Not Predicted." TOB, tobramycin; LEV, levofloxacin; MXF, moxifloxacin; ERY, erythromycin; PEN, penicillin; TET, tetracycline.
